# Talks with Dr. Mandeville

**Published:** 1892-04

**Authors:** 


### TALKS WITH DR. MANDEVILLE.

### No. 3.

Q. Doctor, can you tell me something about the Eye and the Brain, Seeing and Thinking, or the Nervous System?

A. Certainly. I suppose you all remember at a very early time having undergone some such catechism as this: "What is it you walk with?" to which we should reply: "Our legs." "What is it that you strike with?" and we should reply: "Our arms." Or, "What is it that you eat and speak with?" and we should reply: "Our mouths." There are two questions analogous to which similar answers would very likely be given. If we were asked, "What is it that you see with?" we should reply at once: "Our eyes." If we were asked a much more difficult question, "What is that you think with?" we might give two answers, according to what had been told us about it. We might say, "We think with our brains," and that would be an answer analogous to the others. Or we might say: "We think with our minds," and that would be quite a different answer from all the rest. Now, the answer that we should naturally give to the last two questions, viz.,—that we see with our eyes, and think either with our brains or our minds,—are perfectly correct. Either of them is right enough for practical purposes, but neither is perfectly correct; and it is in order to show you what the more correct answer is, which greater knowledge enables us to give, that I want to explain to you something about the mechanism of the brain and that which connects it with the eye. When we have considered this subject completely, we shall find out, I think, that it is not quite correct to say we see with our eyes, and it does not quite accurately represent the facts to say that we think with our brains or even with our minds.

Supposing you see a person's hand resting on the table at tea-time, and that you take a hot spoon out of your tea cup and put it without any warning upon that person's hand; you will find that the hand will jump as soon as the spoon touches it. In the same way, if you suddenly tread upon a person's toe, you will find that the foot by a convulsive movement gets out of the way. A similar fact to that is that if you suddenly and unexpectedly touch a frog, the frog will jump to get out of the way. But there is this difference between the two classes of facts; when you touch the frog with an instrument, and the frog jumps, the sensation is all in the frog; there is nothing outside of the frog which makes him jump. But when you put a hot spoon upon a person's hand, and the hand suddenly jumps away is that all in the hand? Or when you tread upon a man's corn and he suddenly withdraws his foot, is that all in the foot? No, it is not. If you were to cut in two a little white thread which runs up the arm from the hand, you would find that, however hot the spoon was which you applied, the hand would not jump. In the same way, if you cut in two a white thread which runs up the leg, you might tread upon my corns as much as you liked and I should not pull my foot away. It follows from these facts that the connection between the hot spoon and the jumping of the hand, and the connection between treading upon the corn and the motion of the foot are not all in the hand or in the foot, but that there is something else wanted in order to make them complete.

What is that something else? It appears that a message has to be sent away from the hand or the foot to some other part of the body, and another message to come back before a connection can be established between the hot spoon touching the hand and making it jump, or the tread upon the foot making it jump.

Let us consider an analogous case: Suppose a house of business in New York has a branch house at Albany, and the branch house is going to buy goods for the whole firm, but has been instructed not to buy goods without the consent of the principals in New York. Somebody comes with goods to sell to the branch house, and after a little time the branch house buys the goods. Between these two incidents a communication has taken place between the branch house and the central establishment. This communication may be of two kinds. The branch house may have sent down a letter and may have got a letter back through the post-office, or they may have sent a telegram and have got a telegraphic reply. Now, these are two exceedingly different things; they are both messages which are transmitted reciprocally from one point to another, but the mode of transmission in the two cases is essentially different, for in one case a material substance, a letter, carrying in itself a message, travels from the one place to the other, and a similar material substance is sent back; but in the case of the telegram we have very strong reason to believe that there is no material substance made use of. In such case the transmission is not exactly, but very nearly like that which I could produce in a rope stretched from one end of the room to the other. If I give it a shake, that shake travels along the string, but there is no transmission of material substance from one end of it to the other.

Which of those two ways of transmitting a message is used to transmit a message from the hand to some other part of the body, which, as we proceed, we shall find to be the brain, with the effect of making my hand jump when the hot spoon touches it? The mode of transmission resembles more nearly the electric telegraph than the latter; there is no material substance traveling up from my hand to my brain, and back again from my brain to my hand; but there is a certain state of motion which travels along the connections, although it is not exactly the same as that which travels along the telegraphic wire.

How do we know that? We know it by a very simple circumstance, the nerve thread—the white thread which goes from my hand to my brain, is capable of transmitting a message in exactly the same manner as the message transmitted by the electric telegraph wire.

[This subject will be continued in the next number of the JOURNAL.]

